# Reconfigurable Security Architecture (RESA) Based on PUF for FPGA-Based IoT Devices

**DOI:** 10.3390/s22155577

**Published:** 2022-07-26

**Authors:** Armin Babaei, Gregor Schiele, Michael Zohner

**Affiliations:** 1 Nord CS GmbH, 60327 Frankfurt am Main, Germany; ab@nordcs.de; 2 Embedded Systems Department, Duisburg-Essen University, 47057 Duisburg, Germany; 3 Applied Computer Science Department, Hochschule Fulda, 36037 Fulda, Germany; michael.zohner@ai.hs-fulda.de

**Keywords:** physical unclonable function (PUF), IoT, FPGA, authentication, cybersecurity, Internet of Things, security, protocol, partial dynamic reconfiguration

## Abstract

Cybersecurity is a challenge in the utilization of IoT devices. One of the main security functions that we need for IoT devices is authentication. In this work, we used physical unclonable function (PUF) technology to propose a lightweight authentication protocol for IoT devices with long lifetimes. Our focus in this project is a solution for FPGA-based IoT devices. We evaluated the resiliency of our solution against state-of-the-art machine learning attacks.

## 1. Introduction

The Internet of Things (IoT) has become a critical infrastructure of our modern world. It pervades private homes and cities, industrial complexes and offices, farms, hospitals and shopping malls. Due to their critical role, IoT devices have become attractive targets for cyberattacks, which intend to form large-scale bot networks, damage supply chains or even harm users. Hence, cybersecurity is a crucial feature for IoT devices, which, however, is a particularly hard problem, especially due to their (i) low price and (ii) long lifetimes.

IoT devices intrinsically need to be cheap, and thus often use low-cost processing units. Limited resources in these low-cost devices introduce difficulties in modern cybersecurity functions needed for authentication which require cryptographic functions and hence high computational power. To address this issue, scientists in [1,2] introduced a solution based on physically uncloneable functions (PUFs). In this lightweight solution, the design of each hardware module is used to generate a unique fingerprint for each device.

IoT devices—once deployed—must also be usable for many years and always need to have state-of-the-art security measures. If, for example, an IoT device is embedded into a smart vehicle, it must operate for at least 15 years and may even be used for the next 30 years and upwards. In this time, new attacks will be developed, and cryptographic functions considered secure during the design phase of the device may become insecure. PUFs also address this issue, since the security does not depend on characteristics of the function but instead the unique fingerprint of the IoT device.

One solution for providing up-to-date hardware security measures is using FPGA-based designs [3,4]. field programmable gate arrays (FPGAs) are special integrated circuits that can simulate other hardware components, while being re-programmable in the field. Thus, we can use them to implement security measures—like a PUF—very efficiently without losing the ability to update them at any time. This is especially interesting, since many modern devices integrate an FPGA already for complex computations, e.g., for image processing or AI applications [5]. We can use part of the available FPGA space to add a PUF, leaving the rest available for the device’s core functions. Of course, it is important to use up as little space on the FPGA as possible, to minimize our impact on the device’s operation.

The goal of our research is to provide PUF-based lightweight cybersecurity for IoT devices with integrated FPGAs. We extended the Spatial PUF construction of [6], which builds on the dynamic reconfigurability of FPGAs, to provide more challenge response pairs and thereby increase the number of authentication cycles and secure lifetimes of IoT devices. We outline how the Spatial PUF construction can serve as a building block to realize two lightweight authentication protocols, called RESA-P and RESA-PC, which provide security against machine-learning attacks (ML attacks).

### Research Goal Summary

To summarize, we narrow down our research goals to the following points:

1. The authentication procedure based on PUF shall have enough CRPs for IoT product lifetimes. This approach shall not increase the size of the PUF circuit on the chip.
2. The solution shall be compatible with modular system designs on FPGAs (as discussed in Section 2.1).
3. The solution shall have resiliency against machine learning attacks.
4. The proposed solution shall follow the initial promise of PUFs to provide a lightweight security solution without cryptographic primitives.

The paper is structured as follows. In Section 2, we first discuss the concept of PUFs and analyze potential challenges for them, e.g., machine learning attacks. In Section 3, we propose our PUF architecture, which increases the number of CRPs and hence extends the utilization of the PUF for longer product lifetimes. In Section 4, we present our authentication protocols, which we evaluate in Section 5. We conclude the paper in Section 6.

## 2. Background and Related Work

In this section, we give the background on FPGAs and PUFs, evaluate security challenges when implementing PUFs and discuss related work in the respective sections.

### 2.1. FPGAs in IoT Devices

**Figure 1:**
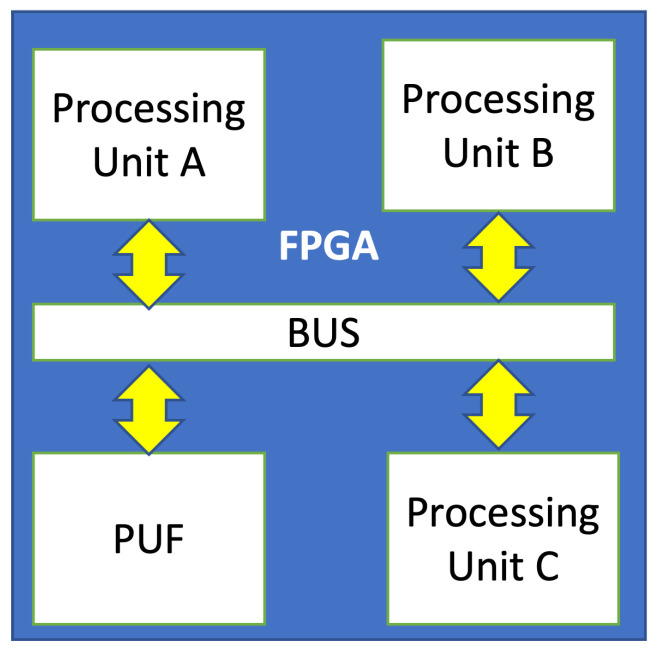
Modular architecture for FPGA-based IoT devices.

IoT devices are very heterogeneous and require different hardware modules for their operation, including special purpose chips for security and communication. To cope with this, embedded FPGAs can instantiate modular systems on chips (SoCs) that are tailor-made for each specific device type. This is shown in Figure 1. In this model there are different processing units instantiated on the FPGA that communicate via an onboard communication bus. Modules can be placed arbitrarily, moved to different positions and updated and replaced with other modules as needed. This can be done offline (using normal FPGA reconfiguration) or online (using dynamic partial reconfiguration [7]). We propose to use a PUF as one module of such a design and to change its placement on the FPGA during system operation.

### 2.2. What Is a PUF?

**Figure 2:**
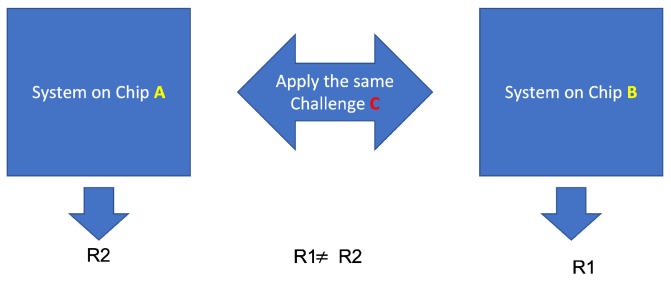
Two identical PUF circuits on two different chips generate different responses.

Due to inaccuracies in the process of manufacturing or assembling, chips of the same product line have small differences in their properties. While these inaccuracies are normally considered an undesirably property, since they lead to inconsistent behavior between devices, we can utilize them in the context of security to achieve individual chip fingerprints. To exploit these, we add a particular circuit design to the hardware, which is known as a PUF circuit (see Figure 2) or PUF for short. PUFs take a chain of bits as an input, namely, a challenge, and create a succession of bits as an output, called the response. For a given challenge, two identical PUFs on different chips produce different responses. We define a challenge–response pair (CRP) as the pair of a challenge and its associated response. A chip’s fingerprint can then be defined as a set of CRPs produced by its PUF.

There are two main types of PUFs, namely, weak and strong PUFs [1], which mainly differ with respect to their relationship between PUF size and the number of available CRPs. In general, a bigger PUF, i.e., a PUF which consumes a bigger area on the die, is able to generate more CRPs. Strong PUFs increase the number of CRPs exponentially with their size. Weak PUFs increase the number of CRPs linearly. As a result, weak PUFs have very limited numbers of CRPs [8] or even only a single CRP, whereas powerful PUFs have massive amounts of CRPs [1]. The focus on our work is on strong PUFs, since we require a high number of CRPs for devices with greatly limited space.

### 2.3. PUF Architecture Selection

As initially discussed, our goal was to develop an authentication procedure that does not require cryptographic primitives. It must be possible to update it, it must fit properly onto an FPGA, and it must be aligned with FPGA characteristics.

The first challenge toward this goal was selecting a PUF architecture that can fit our purposes. Previous work [9] performed an investigation regarding compatibility of PUF architectures on FPGA, and found that ring oscillator (RO) PUFs were the best fit. They are easy to implement on an FPGA, are not path sensitive (as opposed to, e.g., Arbiter PUFs), and can also be used as true random number generators (TRNGs) [10]. Therefore, we selected RO PUFs as the base architecture for our authentication procedure. However, the drawback of the RO PUF architecture is that it has an intrinsic vulnerability [11], to which the authors also provided a solution.

For more details on RO PUFs, we refer the reader to [6,9]. A broader discussion of current FPGA-based PUF architectures can be found in [3].

### 2.4. PUF Applications

**Figure 3:**
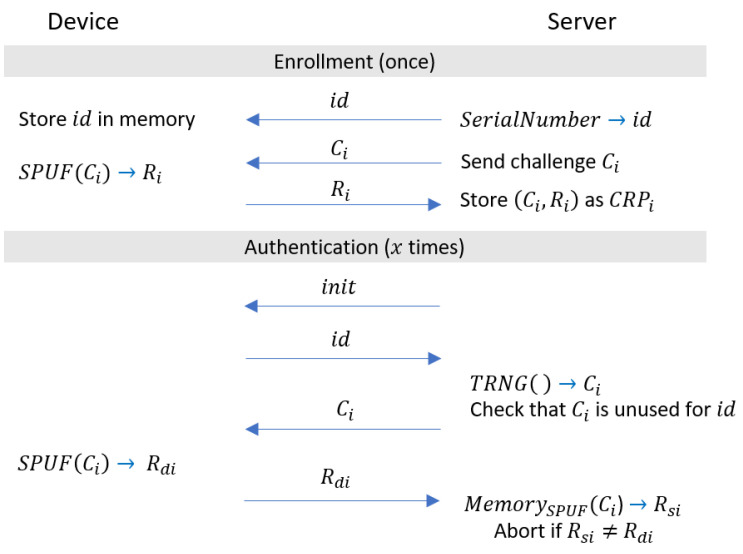
Basic protocol to authenticate a device towards a server using PUFs.

PUFs can be used for key generation and authentication purposes. Our main focus is on authentication of the device towards the server, which is illustrated in Figure 3.

The basic authentication protocol based on a PUF has two operation phases, (1) the enrollment phase and (2) the authentication phase. In the enrollment phase, a server and the device containing the PUF communicate directly in a secure environment. All CRPs are read out and stored securely in the server. Then, later in the authentication phase, the server sends a challenge to the device. The device uses its PUF to generate the response that corresponds to the received challenge and sends it back to the server. If the generated response is identical to the response stored earlier on the server, the device is authenticated.

This is a basic authentication protocol for authenticating the device that is based on a PUF. It does not require any cryptographic primitives (e.g., hashing or encryption) and is lightweight, as only two messages are needed. It should be noted that for each authentication, one unique CRP is required and CRPs cannot be reused. Otherwise, the adversary can simply collect old CRPs and can apply a replay attack on the device. Consequently, weak PUFs cannot be used effectively for this protocol, since they have a rather small number of CRPs. Strong PUFs are better candidates for this application of PUFs [12].

In theory, this basic protocol would be perfect if the CRPs of a PUF are unpredictable. However, in reality, PUFs do not provide the expected resiliency [13] due to various attacks that we review in the following.

### 2.5. PUF Attack Overview

Previous work [9] outlined the cybersecurity challenges in PUFs regarding IoT application. Here we will give an overview to the reader regarding these challenges. In general, there are two types of attacks that are applicable to PUFs. The most effective one are invasive and semi-invasive attacks [9]. In these cases [14], side channel attacks (e.g., electromagnetic analysis, timing, optical, power analysis, etc.) are used to break the PUF security [2]. These attacks are highly effective but require physical access to the attacked device and dedicated equipment.

The second types of attacks are ML attacks [8,15]. These attacks are cost efficient and effective. The attack works by gathering CRPs and modeling them using a ML algorithm such as linear regression in order to predict responses to given challenges. Instead of physical access, an attacker only requires knowledge of some valid CRPs. Currently, ML attacks are the most efficient attacks against IoT devices that use PUF for their authentication process [15]. There are PUF-based protocols and PUF architectures which tackle this challenge. In the following, we will give an overview of them and discuss their applicability to our initially proposed goal, which is to utilize PUFs for FPGA-based devices.

The third types of attacks on PUFs are combinations of side channel attacks with machine learning (ML) attacks [16,17,18]. This attack type is less complex than the previous type but still requires laboratory equipment and physical access to the device, and still might not be cost efficient and applicable for all IoT devices.

### 2.6. PUF Resiliency against ML Attacks

Extensive research has been done to address the vulnerability of PUFs against ML attacks [9]. The first and most straightforward scenario to address ML attacks in is to use hash algorithms to either transmit hashed response values or sign CRPs when transferring them [8,19,20,21]. This is effective but goes against the initial promise of PUFs not to utilize cryptographic primitives.

Researchers also tried to provide resiliency against ML attacks on the hardware level. XOR arbiter PUFs [13] were some of the initially proposed PUF architectures that showed resiliency against ML attacks. Successor architectures, such as [22], also showed resiliency against these types of attacks. Other solutions [23,24] proposed PUF-based authentication protocols that show resiliency against ML attacks. While these solutions provide resilience against ML attacks, they are not available on off-the-shelf hardware. In contrast, our work focuses on providing solutions for resilience against ML attacks based on readily available (embedded) FPGAs on IoT devices.

### 2.7. CRPs vs. Lifetime

One additional challenge remains to be discussed: can PUFs provide secure authentication for the full lifetime of an IoT product? As discussed before, each authentication phase requires (at least) one unique CRP. Thus, we need at least as many CRPs as there are required authentication events during the whole product lifetime. If after some time we run out of CRPs, then we cannot guarantee secure operation anymore. As a consequence, the number of supported CRPs should be as high as possible for a given PUF.

A classic method to increase the number of CRPs is to increase the size of the PUF. By doubling the size of a (strong) PUF, its number of CRPs increases exponentially [6]. However, this approach goes against the original promise of PUFs as a very lightweight security solution. A bigger PUF has a bigger overhead with respect to the consumed chip area and computational power.

**Figure 4:**
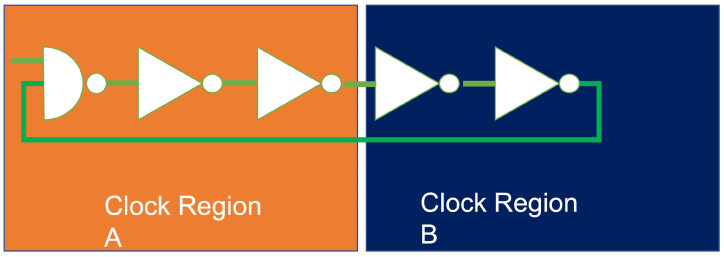
Placing an RO PUF in two different clock regions.

The spatial PUF construction [6] increases the number of CRPs by using multiple PUFs deployed at different clock regions in the FPGA (see Figure 4). The generated CRPs are unique and uncorrelated due to the separation into different clock regions. The original Spatial PUF construction uses a Xilinx XC7A35-T FPGA, which is a tiny FPGA (in term of computational resources) and has six clock regions, which allows increasing the total number of CRPs by a factor of six. Since the goal of our work is to achieve a high number of CRPs without increasing the PUF size, the Spatial PUF approach [6] seems most suitable for our work. We will show in Section 3 how the Spatial PUF construction can be extended to further increase the number of CRPs that can be utilized.

## 3. Extended Spatial PUF

**Figure 5:**
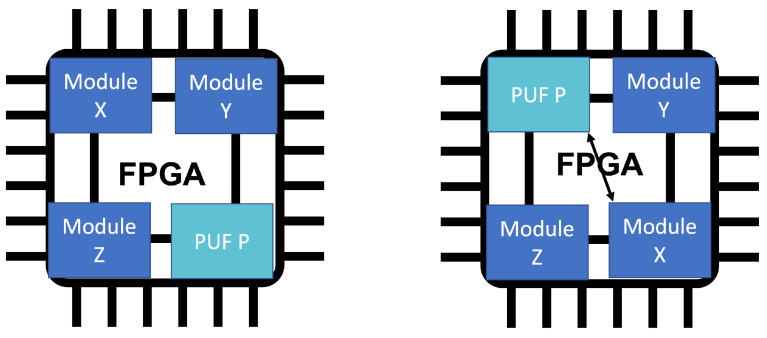
By changing the position of a PUF on an FPGA, we can generate different PUFs with unique and uncorrelated CRPs [6].

In this paper, we extend the Spatial PUF approach of [6] with additional placements. As shown in Figure 5, in addition to placing the PUF in a single clock region, we placed our PUF on the border between two or four different clock regions, leading to 15 different placements. In a border placement between two regions (see Figure 4), the first half of the PUF is placed in clock region A. The second part is placed in clock region B. Our hypothesis is that the difference between clocks in the two clock regions causes different resonating periods of ring oscillators. As a consequence, the resulting PUF will behave differently to PUFs in each of the two clock regions with respect to uniqueness and uniformity.

To examine our hypothesis, we generated all 15 PUF placements, instantiated them on a Xilinx XC7A35-T FPGA (the same FPGA as was used in [6]) and computed the uniformity and uniqueness of the generated CRPs.

*Uniformity* measures whether the distribution of 0 and 1 bits in responses of a single PUF is normalized or not. The optimum value for this measurement is 50%; i.e., the 0 and 1 bits will occur equally often. Therefore, we cannot increase our chances to guess the correct response by favoring either 0s or 1s. The response uniformity at region “*l*” can be calculated as follows:

(1) $$Uniformity=\frac{1}{n}\sum_{i=1}^{n}\left(r_{i,l}\right)*100\%$$

In this formula *i* is the index corresponding to the number of CRPs which is between 1<i<n. We computed this value for all PUFs in the different border regions. The results are presented in Table 1 as distances from the optimal value (50%). The worst case is the PUF in Region BC with 8% distance. The best PUF is the one in Region BCEF with 2% distance. These results are similar to the uniformity rates that were measured for PUFs in single regions in [6].

The other criteria we need to investigate is *uniqueness*. Uniqueness specifies the likelihood of responses of PUFs that are placed in different regions of the FPGA, e.g., comparing the responses of the PUF in Regions D and E with the ones of the PUF in Region DE. We expect that each deployment of our PUF behaves independently. We measure the uniqueness by computing the inter-hamming distance between two deployments l1 and l2 as follows:

(2) $$HD_{inter}=\sum_{l1=1}^{k-1}\sum_{l2=l1+1}^{k}\frac{HD(r_{l1},r_{l2})}{n}*100$$

In this this formula, *k* is the number of regions, in our case 15. Again, the optimal value is 50%, and we show the distance from the optimum in Table 2. The maximum distance is 9%. This is in the acceptable boundary of 10% [1].

In summary, our results confirm our hypothesis that clock regions and border regions act like distinguishable FPGAs with respect to a PUF deployed in them. The generated CRPs are uniform and unique. This allows us to increase the number of CRPs without increasing the size of the PUF on the FPGA, fulfilling our first research goal (see Section 1). The approach is compatible with a modular system design on an FPGA, using the fact that in such a design we can change the placement of the PUF. Thus, we argue that it also fulfills our second research goal. The number of CRPs of a RO PUF with *k* ROs can be calculated as follows:

(3) $$N_{CRP}=\frac{k(k-1)}{2}$$

Thus, a typical RO PUF with k=128 can generate 8128 CRPs. Such a PUF roughly occupies 10% of the used FPGA’s (Xilinx XC7a35T) logic cells. Our technique can increase the number of CRPs by a factor of 15 to 121,920 CRPs, while still only occupying 10% of the FPGA. If we wanted to use a conventional PUF that generates the same amount of CRPs, we would need 512 ROs. This PUF would occupy roughly 40% of the used FPGA.

In the next section, we use our extended Spatial PUF to propose an authentication protocol based on dynamic partial reconfiguration (DPR) technology [7]. This protocol will provide resiliency against ML attacks while providing a lightweight solution, thereby fulfilling our two remaining research goals.

## 4. The RESA PUF Protocols

We now describe how to utilize the Spatial PUF construction to realize lightweight authentication protocols called the Reconfigurable Security Architecture-Based PUF protocols, or RESA for short. We developed two protocol variants that differ with respect to the potential number of CRPs that can be used for authentication and with respect to the security provided. The first protocol, RESA-P, hides the PUF placement in order to allow a full utilization of the CRPs that are accessible from the the Spatial PUF construction. The second protocol, RESA-PC hides the PUF placement and challenge in order to further extend the number of CRPs at the cost of decreasing security over time. Before we present both protocols, we first clarify our underlying assumptions and notations and discuss why an application of the basic authentication protocol from Section 2.4 in the Spatial PUF approach only provides limited security against ML attacks.

### 4.1. Underlying Assumptions and Notation

Our RESA protocols use the Spatial PUF, and we assume that a Spatial PUF is present on the local FPGA of the IoT device that wants to authenticate itself with the server. We further assume that the FPGA supports dynamic partial reconfiguration, as is the case for Xilinx FPGAs [7]. Thus, it can switch between PUF locations without re-configuring the whole FPGA. Our Spatial PUF consists of *M* strong ring oscillator PUFs. We divide the available RO PUFs into two sets: one of static and globally defined RO PUFs, denoted as $SPUF_{ini}$, and M−1 PUF architectures, denoted as $SPUF_{m}$ with m=1...M−1. We denote the total number of CRPs for the Spatial PUF with a RO PUF with *k* paths as $D=M\cdot k\cdot \frac{\left(k-1\right)}{2}$. For common variables between device and server, we distinguish between variables of the client using subscript ${}_{d}$ and variables of the server using subscript ${}_{s}$.

To avoid an attacker fully profiling the device by querying the device with all possible challenges, we assume that the authentication process is time-gated (e.g., only one authentication per day, resulting in 3650 authentications for a span of 10 years). Furthermore, we assume that there is direct physical contact between the device and the server during the authentication, such that man-in-the-middle attacks cannot be performed. Finally, we will omit in our protocol descriptions the handling of the device serial number (denoted id in the basic protocol in Figure 3), since it is identical for all protocols and makes the protocol more complex to read.

### 4.2. A Straightforward Solution

As done in [6], one can directly use the Spatial PUF in combination with the basic authentication protocol shown in Figure 3. This is done by reading out all CRPs for all placements in the enrollment phase. In the authentication phase, the server sends the challenge and the placement it wishes to query the challenge on. Thereby, the server can cycle through all challenges and placements in the authentication phase to utilize all available CRPs.

However, as we discussed earlier and as we will show in Section 5.3, PUFs are vulnerable to ML attacks in which the adversary gathers CRPs and uses them to model the PUF and predict responses to unused challenges. These ML attacks carry over to the straightforward authentication protocol for the Spatial PUF construction, since the attacker can infer the placement from the message sent from server to device and treat each placement as a different PUF that he can attack in isolation. Hence, even tough each placement will be secure for some authentication cycles, it is only a question of time until the attacker gathers sufficient CRPs to create an accurate model. The exact number of CRPs that is needed for this depends on the concrete ML technique that is used in the attack.

A simple fix to counter this attack is to not use all CRPs for each placement, but instead to avoid invoking the PUF placement once a certain threshold of CRPs has been used. This simple fix, however, has several drawbacks:

- Research on ML attacks continuously reduces the numbers of CRPs required to accurately model a PUF [25]. Hence, once a threshold is set in practice, there is no guarantee that this threshold will remain secure in the future.
- If we select the threshold to a low value, we would lose many of the available CRPs and would thereby greatly reduce the operational lifetimes of the devices.
- An attacker may query the device on certain placements to model the PUF independently of genuine authentication requests by the server. While the impact of such a scenario could be reduced by time gating mechanisms, the attacker would still be able to perform some queries and thereby bypass the threshold.

Thus, we require a more sophisticated authentication protocol that provides protection against ML attacks.

### 4.3. RESA-P Authentication Protocol

As we have discussed, utilizing the Spatial-PUF architecture in a straightforward authentication protocol does not provide security against ML attacks, since each PUF architecture can be modeled independently. The key aspect that allows an attacker to model the PUFs independently is that the server sends the placement in its query towards the device. By hiding the placement from the attacker, we could prevent the attacker from modeling the PUFs independently, and hence increase the problem of learning the underlying PUF model to a multi-dimensional problem that is harder to solve for ML attacks. There are several possibilities for hiding the placement which we ruled out due to our context of long-term authentication for IoT devices with low resources:

- Encrypting the placement: Not possible, since we want to avoid cryptographic functions, which may become insecure over time.
- Fixing an order during enrollment: Not possible, since the order (e.g., a seed of a PRNG or sequential list of placements) is confidential and needs to be stored in a dedicated secure memory, which may not be available on low resource devices.

**Figure 6:**
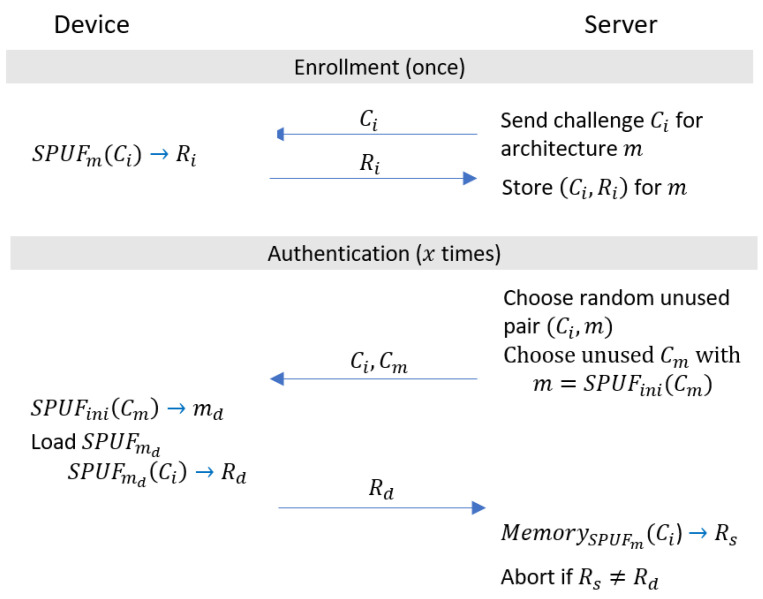
Our RESA-P authentication protocol, which hides the PUF architecture *m*, from which the CRP $\left(C_{i},R\right)$ for authentication is used.

Instead, in order to hide the placement, we make use of the resources that we have available on the device, namely, PUFs. More concretely, the server hides the placement *m* by sending a challenge $C_{m}$ that is evaluated on a fixed PUF placement $SPUF_{ini}$ as $m=SPUF_{ini}\left(C_{m}\right)$. Since the attacker does not know the model of $SPUF_{ini}$ or receive the resulting placement *m*, it is not possible for them to correlate the CRPs used for authentication to the individual PUF placements. In the following, we describe our protocol RESA-P for hiding the placement, which is displayed in Figure 6, in more detail.

#### 4.3.1. Enrollment Phase

The enrollment phase is analogous to the straightforward protocol in Figure 3, except that the server queries the device for all CRPs of all *M* PUF architectures.

#### 4.3.2. Authentication Phase

In the authentication phase, the server randomly selects a CRP with challenge $C_{i}$ from an architecture *m*, which together have not already been used for authentication. In the next step, the server needs to hide the selected architecture *m* using a challenge $C_{m}$ that will be evaluated on the fixed architecture $SPUF_{ini}$ with $m=SPUF_{ini}\left(C_{m}\right)$. Observe that we require a reverse lookup, since we fix *m* and choose $C_{m}$ adequately. We argue that this step is possible, since the CRPs of $SPUF_{ini}$ are known to the server, and it can build the challenge $C_{m}$ bit-wise using single CRPs. In addition, since the 0s and 1s in the responses of the Spatial PUFs are uniformly distributed, there exist, with high probability, sufficient CRPs from $SPUF_{ini}$ that can be used to build the challenge $C_{m}$.

The server then sends both challenges $C_{i}$ and $C_{m}$ to the device. The device queries the initial PUF $SPUF_{ini}$ on the architecture challenge $C_{m}$ to obtain the architecture to be loaded $m_{d}=SPUF_{ini}\left(C_{m}\right)$. It then partially re-configures itself to load the architecture $SPUF_{m_{d}}$, which it queries on $C_{i}$ to obtain the response for the authentication process $R_{d}=SPUF_{m_{d}}\left(C_{i}\right)$, which it sends to the server. The server verifies the authentication response by looking up from memory the stored response $R_{s}$ for challenge $C_{i}$ and architecture *m* as $R_{s}=Memory_{SPUF_{m}}\left(C_{i}\right)$. The device is successfully authenticated if $R_{d}$ equals $R_{s}$, and otherwise, the server aborts the protocol.

#### 4.3.3. Security Proof

The goal of our RESA-P protocol is to authenticate the device towards the server. We assume that an attacker is able to eavesdrop on all messages $C_{i}$, $C_{m}$ and $R_{d}$ used for all previous genuine authentication protocols, but does not know the models $SPUF_{m}$ or $SPUF_{ini}$ which were used to calculate the responses. In addition, the attacker is able to query the device on a limited number of challenges $C_{i}$ and $C_{m}$ for which it receives the response $R_{d}$. The goal of the attacker is to authenticate himself better than with probability $2^{-\ell_{R}}$ (which the attacker can always do by guessing $R_{d}$) by providing the correct response $R_{d}=R_{s}$ for challenges chosen by the server $C_{i}$ and $C_{m}$.

We first consider the information that an attacker can learn from observing a genuine protocol execution. The attacker learns the CRP $\left(C_{i},R_{d}\right)$ and $C_{m}$ but does not learn the architecture *m* of the CRP. This is due to (1) the PUF architecture $SPUF_{ini}$ being unknown to the attacker, (2) the challenge $C_{m}$ never being re-used and (3) the architecture *m* not being communicated outside of the device or server. Modeling the internal PUF $SPUF_{ini}$ is not possible for an attacker, since he never receives the response $m=SPUF_{ini}\left(C_{m}\right)$ for a given challenge $C_{m}$. Finally, performing a ML attack on only the CRPs, without distinguishing them by architecture, is not possible, as we will demonstrate empirically in Section 5.4.

Next, we consider the case in which the attacker replays for a specific challenge the response $R_{d}$. Observe that the server chooses the challenges from unused CRPs and architectures, and hence, the server cannot replay responses from genuine protocol executions. Other than from genuine protocol executions, the attacker could have the device’s generate valid responses $R_{d}$ by querying on the respective challenges $C_{i},C_{m}$. Note that the attacker is only able to generate these valid responses for a limited number, *t*, of challenges. Hence, with *D* CRPs remaining, there is only a chance of $t\times 2^{-D}$ that the attacker correctly pre-generated the corresponding response. While this chance may be high when few authentication requests have been performed by the server, it gradually decreases with the number of authentication requests performed. In this worst case, namely, when only one CRP on one architecture is available to the server, the attacker may pre-generate the response with probability $\frac{1}{M-1}$, since the architecture is unknown to him.

There are several strategies with which to avoid this attack. Firstly, one could not utilize all CRPs to reduce the probability that an attacker successfully will pre-generate the correct response. Alternatively, one could count the number of authentication requests on the device and stop giving a response once a certain threshold has been reached. Finally, we point out that our protocol could be extended similar to PUF protocol constructions of [23], where the server provides some form of proof to obtain the response from the device.

#### 4.3.4. Re-Using CRPs

Based on the fact that the PUF architecture *m* of a CRP remains hidden, the question arises of whether it is possible to re-use CRPs in combination with different architecture challenges $C_{m}$ in order to extend the number of authentication processes. We answer this question in the negative, as re-using CRPs drastically reduces the security of our RESA-P protocol.

Consider the case where all challenges $C_{i}$ have been used and are known to the attacker but the architecture challenges $C_{m}$ are not re-used. Hence, for each challenge $C_{i}$, the attacker knows all *M* possible responses $R_{i}$ for each of the architectures, but cannot assign these responses to a particular architecture. When a new authentication process is then triggered, the attacker can guess the architecture *m* and authenticate himself successfully with probability $\frac{1}{M-1}$.

Next, consider the case where the architecture challenge is re-used $C_{m}$ between two authentication processes with CRPs $\left(C_{i},R_{d}\right)$ and $\left(C_{i^{\prime }},R_{i^{\prime }}\right)$. In this case, the attacker knows that both CRPs, $\left(C_{i},R_{d}\right)$ and $\left(C_{i^{\prime }},R_{i^{\prime }}\right)$, were from the same PUF architecture *m*. This allows an attacker to train a ML model for a PUF *m*, rendering the protocol insecure. We will show in the next section on our RESA-PC protocol, how CRPs can be re-used without sacrificing too much security.

### 4.4. RESA-PC Authentication Protocol

Our RESA-P protocol does not allow the re-use of CRPs, since CRPs can be distinguished by their architectures, and already observed responses can be replayed. These downsides are due to two problems:

1. The challenge $C_{i}$ of the CRP is communicated in the open;
2. There can only be $2^{D}$ possible CRPs, which the attacker can infer by observing the challenges communicated by the server.

**Figure 7:**
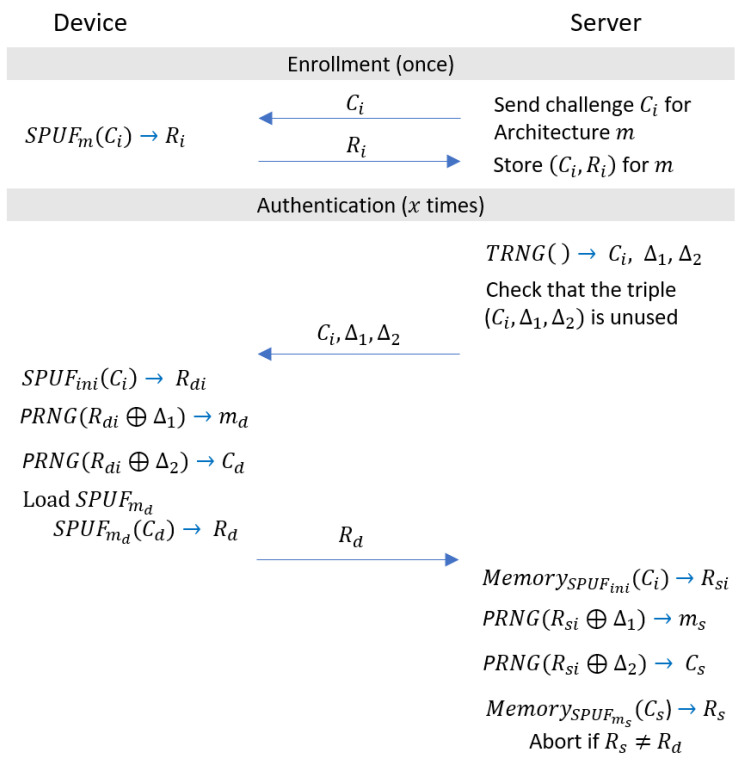
Our RESA-PC authentication protocol, which hides the PUF architecture *m* and challenge $C_{i}$, which is used for authentication.

In the following, we will describe how to solve these two problems, which led to the design of our RESA-PC protocol (RESA with hidden placement and challenge), described in Figure 7.

For the solution to the first problem, the challenges being communicated in the open, notice that we can apply the same insight as in the design of our RESA-P protocol. Concretely, we can hide the challenge of the CRP by applying the challenge $C_{i}$ to the initial PUF $SPUF_{ini}$, thereby gaining an intermediate challenge $C_{d}=SPUF_{ini}$, which is hidden from the attacker. This yields the advantage that the attacker is not able to correlate the challenge $C_{d}$ and corresponding response $R_{d}$. However, this also brings the disadvantage that we require many CRPs from $SPUF_{ini}$ to generate the intermediate challenge $C_{d}$ in addition to the architecture *m*. To reach a high number of authentication attempts, we would need to re-use CRPs from $SPUF_{ini}$. An attacker could thereby observe the re-use of CRPs and exploit them by inferring information on internal values $C_{d}$ and *m*.

Next we show how to solve this and the second problem, namely, that a re-use of internal CRPs can be observed from the challenges sent by the server. Informally, a re-use of CRPs is difficult to prevent, since the possible combinations for the challenges sent by the server are capped by the maximum number of CRPs. This maximum number is difficult to increase, since it is bound by physical properties of our device. To overcome this problem, we introduce two additional random values, $\Delta_{1}$ and $\Delta_{2}$, which are used to decouple the values of the internal challenge $C_{d}$ and the architecture *m* from the challenge $C_{i}$ sent by the server. In the following, we describe our RESA-PC protocol in more detail.

#### 4.4.1. Enrollment Phase

The enrollment phase is the same as RESA-P.

#### 4.4.2. Authentication Phase

The server first generates the challenge $C_{i}$ and two values, $\Delta_{1}$ and $\Delta_{2}$, using a true-random number generator (TRNG), checks that they have not been used for an authentication attempt before and sends them to the device. In the next step, the device evaluates $SPUF_{ini}$ using the received challenge $C_{i}$ to obtain its internal response $R_{di}=SPUF_{ini}\left(C_{i}\right)$. This internal response $R_{di}$ is used as base to calculate the architecture $m_{d}=PRNG\left(R_{di}⊕\Delta_{1}\right)$ and the internal challenge $C_{d}=PRNG\left(R_{di}⊕\Delta_{2}\right)$. The PRNG can be instantiated using a non-linear PRNG construction with uniform distribution to avoid the exploitation of linear correlations. The device then proceeds as in the RESA-P protocol: It partially re-configures itself to load $SPUF_{m_{d}}$, calculates $R_{d}=SPUF_{m_{d}}\left(C_{d}\right)$ and sends $R_{d}$ to the server. The server then mirrors the steps of the device using its internal memory, resulting in $R_{s}$, authenticates the client if $R_{s}$ is equal to $R_{d}$ and aborts otherwise.

#### 4.4.3. Security Proof

We sketch the security proof of the RESA-PC protocol in the same model as the RESA-P protocol. We assume that an attacker wants to impersonate the device, and can eavesdrop the messages $C_{i},\Delta_{1},\Delta_{2}$ and $R_{d}$ of all genuine authentication protocol executions but does not know the models $SPUF_{m}$ and $SPUF_{ini}$. In addition, the attacker can query the device on a limited number of challenges $C_{i},\Delta_{1},\Delta_{2}$ for which he receives the response $R_{d}$.

From a genuine protocol execution, the attacker learns $C_{i},\Delta_{1},\Delta_{2}$ and $R_{d}$, but does not get information on the internal response $R_{di}$, the PUF architecture for the challenge $m_{d}$ or the internal challenge $C_{d}$. The attacker cannot learn information on $R_{di}$, since he does not know the PUF model $SPUF_{ini}$, based on which it is calculated. Furthermore, since the values $R_{di}$ of other protocol executions are not leaked to the attacker, he is not able to correctly model $SPUF_{ini}$. The values $m_{d}$ and $C_{d}$ are not known to the attacker, since (1) the value $R_{di}$, based on which they are calculated, is also not known to the attacker and (2) it is not possible to infer the challenge $C_{d}$ from the response $R_{d}$ due to the security features of the PUF. Hence, the only values that an attacker can correlate are $C_{i},\Delta_{1},\Delta_{2}$ against $R_{d}$. The attacker is only able to guess the correct response $R_{d}$, in case he has already recorded an authentication execution for the same triple $C_{i},\Delta_{1},\Delta_{2}$, since changing either of these three values will likely result in a different $R_{d}$ (note that the response $R_{d}$ only “likely” changes, since changes in $\Delta_{2}$ result in the same architecture being chosen with probability $\frac{1}{M-1}$).

We require the PRNG to be non-linear and produce a uniformly distributed output in order to avoid an attacker exploiting linear correlations between inputs or tendencies towards certain outputs. Learning the underlying models using ML attacks is also difficult, as we empirically verify in Section 5.4, since there are two PUF architectures involved in computing the response $R_{d}$, from which one is randomized and unknown to the attacker.

Other than observing protocol executions, the attacker could input its own choice of $C_{i},\Delta_{1},\Delta_{2}$ that are specifically chosen to learn information on the internal models $SPUF_{m}$ or $SPUF_{ini}$. For instance, he could fix two values from $C_{i},\Delta_{1},\Delta_{2}$ and iterate over the third. By fixing $\Delta_{1}$ and $\Delta_{2}$ and iterating over $C_{i}$, he could classify different challenges $C_{i}$ by their internal responses $R_{di}$, albeit not being able to learn the actual value of $R_{di}$. This can be exploited by an attacker as follows. An attacker that has classified two challenges, $C_{i}^{a}$ and $C_{i}^{b}$, with $C_{i}^{a}\neq C_{i}^{b}$ as having the same internal $R_{di}$ could exploit this knowledge if he has recorded an authentication execution on $C_{i}^{a},\Delta_{1}^{a},\Delta_{2}^{a},R_{d}^{a}$. In case the server sends the values $C_{i}^{b},\Delta_{1}^{b},\Delta_{2}^{b}$ with $\Delta_{1}^{a}=\Delta_{1}^{b}$ and $\Delta_{2}^{a}=\Delta_{2}^{b}$, which is an unused value, since $C_{i}^{a}\neq C_{i}$, the attacker can reply with a correct $R_{d}$. Varying the value $\Delta_{1}$ (or $\Delta_{2}$) and fixing the respective other two, provides similar potential for classifying the results based on the architecture (or the challenges). In order to protect against these types of classification attacks, we need to choose values $C_{i},\Delta_{1},\Delta_{2}$ which are sufficiently large to decrease the probability that two values of $C_{i},\Delta_{1},\Delta_{2}$ between different protocol executions are actually chosen to be the same. By choosing the size of each value as 40 bit, one could reduce this chance to $2^{-80}$.

#### 4.4.4. Re-Using CRPs

Other than for our RESA-P protocol, we argue that it is possible in our RESA-PC protocol to re-use CRPs. Consider the case where all CRPs have been used for authentication once and the attacker knows the responses $R_{d}$ of all CRPs. We argue that an attacker is not able to take advantage of this knowledge, as long as he does not have information on the internal SPUF models. The reason is that the response $R_{d}$ depends on all three values $C_{i},\Delta_{1}$ and $\Delta_{2}$, and for unused combinations of these values, the attacker is not able to infer additional information from already observed executions (except with some negligible probability in the bit-length of $C_{i},\Delta_{1},\Delta_{2}$, as discussed previously).

Note, however, that this argumentation only holds as long as the attacker is not able to derive information on the internal SPUF models. We stress that it is possible for an attacker to infer internal information in a case where, e.g., the same $R_{d}$ is observed. In this case, the attacker can infer that the same architecture *m* and internal challenge $C_{di}$ were used. These information leaks, as minor as they may seem, allow an attacker to model the internal SPUF models and hence break the security of the protocol. Quantifying the theoretical information leak exactly, however, is a challenging task that is outside of the scope of this paper. Instead, we utilize empirical measures in the following to obtain an understanding on the level of this information leak.

## 5. Evaluation

The main reason behind developing the RESA protocols was to provide resiliency against ML attacks for PUF-based authentication. In this section, we evaluate to what extent our RESA protocols fulfill this, i.e., how resilient they are against state-of-the-art ML attacks. To do so, we introduce our test strategy for the empirical evaluation, validate it and present the evaluation results.

### 5.1. Test Strategy

Our initial approach to evaluate our protocol’s resiliency against ML attacks was to develop and apply a set of existing ML attacks and then analyze whether our protocol is resilient against them or not. However, this way we could only prove whether the developed protocol is resilient against the specific set of ML attacks we use. We could not perform an assessment of its general resiliency against ML attacks. An alternative approach would be to evaluate measurable quantified factors of the protocol that define the resiliency of the protocol against ML attacks. This approach is provided by PUFmeter, which we decided to use for our evaluation.

PUFmeter is an open source toolbox for evaluating the resiliency of real-world PUFs against ML attacks with no focus on any specific PUF architecture [25]. It was developed by Fatemeh Ganji [25] and can be found on the Trust-Hub platform (https://trust-hub.org/home, accessed on 20 June 2022). PUFmeter has to be fed a large number of CRPs to create a model to assess the quality of a PUF in terms of learnability. A point to note is that PUFmeter sees a PUF simply as mapping from a set of challenges (i.e., inputs of the PUF) C to a set of responses R which are the outputs of the PUF, i.e., $f_{PUF}\left(C\right)=R$.

The idea behind the tool is to represent a PUF by Boolean equations over a finite field and attempt to derive information about the challenge–response behavior of a PUF. This mapping is determined by the inherent physical characteristics of the device embodying the PUF [25]. The PUFmeter can address the question of to what degree a PUF can be modeled by applying ML attacks.

PUFmeter employs provable techniques rather than relying on trial and error, allowing a user to decide how accurate the results should be and with what level of certainty the results should be produced. The details of this implementation are discussed in [25].

### 5.2. PUFmeter Testing Procedure

**Figure 8:**
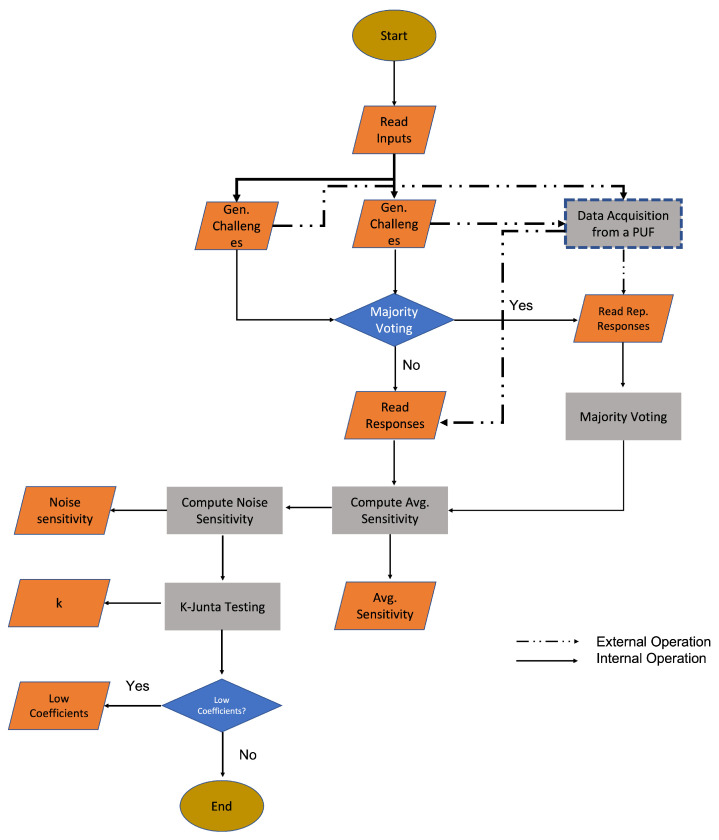
The workflow of PUFmeter [25].

The whole testing procedure used by PUFmeter is illustrated in Figure 8. The algorithm first reads in configuration data, e.g., the number of bits *n* in a challenge (which reflects the maximum possible number of CRPs) and the desired confidence level of the test. The latter can be used to specify how many of the possible CRPs are actually tested.

After this step, PUFmeter generates challenges according to the received inputs. These challenges will be applied to the PUF, and the PUF will generate its responses. Note that PUF CRPs are noisy. To cope with this, PUFmeter applies each challenge multiple times to the PUF and uses majority voting on the results to generate CRPs. We skipped this noise-reduction step by including the technique of [2] in our PUF implementation, which reduced the noise level of CRPs.

The next step is to compute the “average sensitivity”. Average sensitivity determines the influence of challenge bits on the responses. If the value of the average sensitivity decreases, the probability that a ML attack will be able to predict responses increases.

Let us consider the following real-valued Boolean function f on the domain $:{\left\{-1,1\right\}}^{n}\to R$. The ith influence of a function is given by ${\mathrm{Inf}}_{i}\left[f\right]=\mathrm{Pr}\left[f\left(C\right)\neq f\left(C{}^{⊕}i\right)\right]$, where $C{}^{⊕}i$ is obtained by flipping the *i*th bit of C.

If f is Boolean, then ${\mathrm{Inf}}_{i}\left[f\right]$, or the ith influence of a Boolean function is the probability that flipping the ith coordinate flips the value of the function. If ${Inf}_{i}\left[f\right]=0$, then f does not depend on the *i*th coordinate. The total influence of f is the sum of all of its influences:

$$Inf\left[f\right]=\sum_{i=1}^{n}{Inf}_{i}\left[f\right]$$

The next factor that PUFmeter computes is “noise sensitivity”. This determines the effect of flipping a bit in the challenge on the response. This “noise sensitivity” should not be confused with noisy CRPs, which we discussed previously.

In the case of noise sensitivity, given the Boolean function

$$f:{\left\{-1,+1\right\}}^{n}\to \{-1,+1\},$$

if we assume C to be a string chosen randomly and uniformly, then by flipping each bit of C independently with a probability of ε between 0 and 1 (0≤ε≤1), we obtain the flipped string $C^{{}^{\prime }}$. The noise sensitivity of the Boolean function f at that probability ε is given by

$$NS\varepsilon \left(f\right):=(Pr\left[f\left(c\right)\neq f\left(c^{{}^{\prime }}\right)\right])$$

The next step in PUFmeter is “K-junta Testing”. K-junta testing algorithms are well-studied algorithms for property testing. Generally, an ML algorithm learns a class of target functions, and the property tester examines whether the property of a specific function can be found in the function under test or not. More specifically, in the context of a PUF, the property testing algorithm examines whether it is possible to approximate the function f(C)=R with a class of Boolean functions from a specific class. Therefore, PUFmeter can use K-junta algorithms to figure out whether the function f(C)=R can be approximated or not.

If K-junta testing is successful, then the final step in the PUFmeter is to compute the so-called “low coefficients”, a small subset of Boolean variables positioned in the lower part of a Fourier spectrum. The main idea behind this is that a Fourier transform can adequately represent a Boolean function. Linial et al. [26] showed the correlation between a Fourier spectrum and learnability. The main result of their research is that the “low coefficients” are enough to approximate Boolean functions (e.g., K-junta functions). In the context of PUFmeter, the low-degree algorithm has been implemented to approximate the PUF function f(C)=R by determining the most dominant Fourier coefficients of this function. The output of this part is the low coefficients of the PUF function f(C)=R.

All of the extracted factors and their effects on the learnability of a PUF are shown in Table 3.

### 5.3. PUFmeter Validation

Before evaluating our own approach, we first evaluated whether PUFmeter can reliably detect that a PUF is vulnerable to ML attacks. To do so, we decided to test PUFmeter with a ring oscillator (RO) PUF, similarly to the study done by Ganji et al. [25]. This way we can also compare it to our spatial PUF later, which internally uses RO PUFs, too. Ruhrmair et al. [8] previously proved that RO PUFs are vulnerable to ML attacks. They applied empirical ML algorithms to show this.

For our validation, we used an RO PUF with 256 ring oscillators, which generates 8128 CRPs. We configured PUFmeter to use 40% of the CRPs. The result is shown in Table 4. As can be seen, both the average sensitivity and the noise sensitivity are comparatively low. Accordingly, K-junta testing found that the RO PUF only has two influential bits (K=2), not enough to be robust against ML attacks. Finally, K-junta testing was successful and found two low-valued coefficients. Overall, this shows that an RO PUF is vulnerable to ML attacks. We repeated the test on four different FPGA boards with identical PUF architectures. In all scenarios, the PUFmeter showed similar results.

Our validation test showed that PUFmeter works as expected, and we decided to use it to evaluate our protocol.

### 5.4. Evaluating the RESA PUF Authentication Protocol

After validating our testing tool, we used PUFmeter to evaluate the robustness of our RESA-P and RESA-PC protocols. We decided to check the resiliency of our protocol by varying the given number of CRPs. More specifically, we wanted to know: “how many CRPs are required for the ML attack to be effective, such that it can predict the rest of the CRPs?” For this purpose, we varied the number of CRPs that the PUFmeter used to attack our approach. In total, we had 48768 CRPs. We applied between 40% and 100% of the CRPs to find the breaking point of our protocol. The results of this test are shown in Table 5.

**Figure 9:**
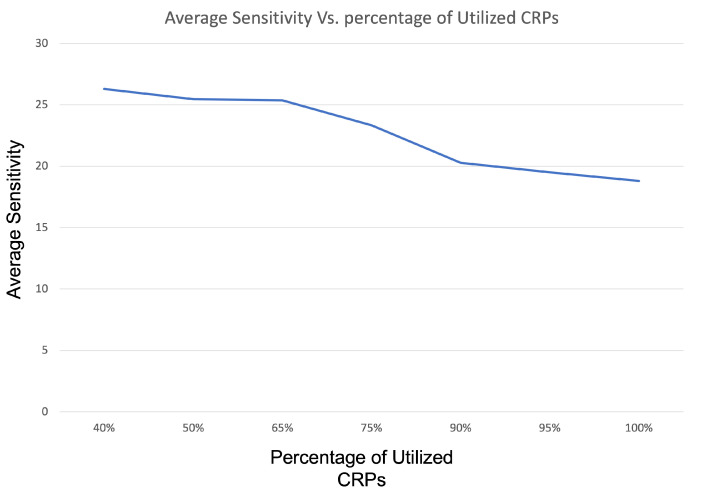
Average sensitivity behavior by increasing the number of CRPs.

As we can see, the average and noise sensitivities are higher than for the RO PUF. The noise sensitivity remained almost constant at around NF(f)≈0.24. The average sensitivity decreased for higher percentages of provided CRPs. To look at this a bit closer, we refer to (Figure 9). Initially, up until approximately 65% of the CRPs, the average sensitivity was quite stable. After that, it decreased linearly, until it reached approximately 18.8 for 100% CRPs. Importantly, for all cases, the K values stayed constant at 13. Thus, although an attacker can make some potential progress with respect to learning the PUF behavior the high value of K means that K-junta testing is not successful in modeling the system. This is why no coefficients are provided.

We repeated this test on four different sets of CRPs which were collected from four different FPGA boards. In all cases the K-junta algorithm was not successful and LMN coefficients were not created by PUFmeter. According to the result of our tests, we can conclude that our RESA PUF authentication protocol is resilient against state-of-the-art ML attacks, even for cases in which a large percentage of the CRPs are intercepted by an attacker. Note that this applies only to current ML attacks. We do not claim that our protocol is resilient against ML attack scenarios that will be developed in the future.

## 6. Conclusions and Future Work

In this paper, we proposed two authentication protocols, called RESA-P and RESA-PC, for FPGA-based IoT devices with long lifecycles. Our protocols do not utilize cryptographic primitives (e.g., hashing or encryption) and are resilient against state-of-the-art machine learning attacks As a build block, we used the Spatial PUF architecture, which allows us to co-locate a PUF with other components in a SoC. During run-time, the location of the PUF is changed dynamically on the FPGA, leading to independent PUFs that we can switch between. Our design so far is based on a ring oscillator PUF.

The current solution shows resiliency against state-of-the-art machine learning attacks. These attacks can be made with minimum hardware requirements and remotely. Other attacks with more complex needs (e.g., a combination of side-channel and machine learning, hardware-based, DoS, etc.) were out of scope. As the next step for this work, the resiliency of this protocol needs to be tested against other attack scenarios, and further improvement might be required to level up the resiliency of the protocol against these attacks.

In future work, we plan to integrate our Spatial PUF into a SoC solution for IoT devices to evaluate its practical usability in real deployments and application scenarios. We also want to extend our approach to ensure integrity and confidentiality. As initially discussed, the RESA-PC protocol that reuses CRPs needs further investigation. The state-of-the-art ML-attack test platform of [25] was not able to successfully break the protocol. This may be largely due to non-fitting models of the ML-attacks, which require extensive and tailor-made adaption towards the RESA-PC protocol.

## Figures and Tables

**Table 1:** Distribution of 0s and 1s in response bits at each border region and their distances from the optimal value.

	Region AB	Region AD	Region ABDE	Region BE	Region BC	Region BCEF	Region CF	Region EF	Region DE
Distance from Opt. Value	5%	3%	4.5%	6%	8%	2%	7%	3.5%	4%

**Table 2:** Distance from $HD_{inter}$ optimum value between i and j border regions or clock regions of FPGA.

	Region A	Region B	Region C	Region D	Region E	Region F	Region AB
Region A							
Region B	1%						
Region C	0%	2%					
Region D	8%	8%	2%				
Region E	4%	3%	1%	6%			
Region F	1%	5%	1%	5%	3%		
Region AB	6%	7%	5%	3%	2%	4%	
Region AD	5%	3%	1%	4%	7%	2%	6%
Region ABDE	7%	4%	8%	2%	5%	1%	3%
Region BE	2%	3%	5%	0%	4%	7%	1%
Region BC	1%	1%	7%	9%	4%	5%	5%
Region BCEF	4%	4%	9%	4%	2%	8%	3%
Region CF	8%	5%	3%	2%	4%	1%	5%
Region EF	3%	7%	8%	2%	6%	8%	5%
Region DE	4%	2%	0%	7%	8%	5%	6%
	Region AD	Region ABDE	Region BE	Region BC	Region BCEF	Region CF	Region EF
Region AD							
Region ABDE	7%						
Region BE	3%	6%					
Region BC	7%	3%	9%				
Region BCEF	6%	8%	1%	6%			
Region CF	3%	2%	1%	7%	5%		
Region EF	6%	5%	4%	8%	3%	9%	
Region DE	7%	4%	1%	8%	5%	6%	1%

**Table 3:** Notion and techniques that are introduced and used in PUFmeter.

Metrics & Techniques	Description	Interpretation	Theoretical Bound
Average sensitivity (*p*)	Average influence of challenge bits on the responses (over all challenge bits)	Smaller values correlate to higher probability of learning	I(f)>>p(ln(n))
Noise Sensitivity ($NS_{\epsilon }$)	Total impact of flipping bits (with probability ϵ) on the responses	Smaller values correlate to higher probability of learning	$NS_{\epsilon }\left(f\right)>>\epsilon^{1/2}$
K-junta Testing (*k*)	Number of influential bits	Smaller values correlate to higher probability of learning	k>>p(ln(n))
LMN Algorithm	Finding a small portion of the Fourier spectral determining the responses	Fewer and smaller coefficients correlate to higher probability of learning	N/A

**Table 4:** PUFmeter results on RO PUF vulnerability against ML attacks.

Average Sensitivity	Noise Sensitivity	K-Junta Determinant Variables	Coefficients	Conclusion
5.694882	0.1961122	2	0.73529, 0.32353	PUF is vulnerable to K-junta Testing

**Table 5:** PUFmeter test results on the resiliency of the RESA authentication protocol.

Percentage CRP	Number of CRPs	Average Sensitivity	Noise Sensitivity	K-Junta Determinant Variable
40	19,508	26.292	0.24544	13
50	24,384	25.455	0.24151	13
65	31,700	25.367	0.24539	12
75	36,576	23.337	0.24661	13
90	43,892	20.284	0.2467	13
95	46,330	19.496	0.24738	13
100	48,768	18.804	0.24701	13
